# Does Inhibitory Repetitive Transcranial Magnetic Stimulation Augment Functional Task Practice to Improve Arm Recovery in Chronic Stroke?

**DOI:** 10.1155/2014/305236

**Published:** 2014-08-13

**Authors:** Dorian K. Rose, Carolynn Patten, Theresa E. McGuirk, Xiaomin Lu, William J. Triggs

**Affiliations:** ^1^Department of Physical Therapy, College of Public Health and Health Professions, University of Florida, P.O. Box 100154, Gainesville, FL 32610-0154, USA; ^2^Brain Rehabilitation Research Center, Malcom Randall VA Medical Center, Gainesville, FL, USA; ^3^Department of Applied Physiology and Kinesiology, University of Florida, Gainesville, FL, USA; ^4^Department of Neurology, University of Florida, Gainesville, FL, USA; ^5^Department of Biostatistics & Children's Oncology Group, University of Florida, Gainesville, FL, USA

## Abstract

*Introduction*. Restoration of upper extremity (UE) functional use remains a challenge for individuals following stroke. Repetitive transcranial magnetic stimulation (rTMS) is a noninvasive modality that modulates cortical excitability and is being explored as a means to potentially ameliorate these deficits. The purpose of this study was to evaluate, in the presence of chronic stroke, the effects of low-frequency rTMS to the contralesional hemisphere as an adjuvant to functional task practice (FTP), to improve UE functional ability. *Methods*. Twenty-two individuals with chronic stroke and subsequent moderate UE deficits were randomized to receive 16 sessions (4 times/week for 4 weeks) of either real-rTMS or sham-rTMS followed by 1-hour of paretic UE FTP. *Results*. No differences in UE outcomes were revealed between the real-rTMS and sham-rTMS intervention groups. After adjusting for baseline differences, no differences were revealed in contralesional cortical excitability postintervention. In a secondary analysis, data pooled across both groups revealed small, but statistically significant, improvements in UE behavioral measures. *Conclusions*. rTMS did not augment changes in UE motor ability in this population of individuals with chronic stroke. The chronicity of our participant cohort and their degree of UE motor impairment may have contributed to inability to produce marked effects using rTMS.

## 1. Introduction

Between 55% and 75% of stroke survivors experience limitations in functional use of the upper extremity at six-months post-stroke [1]. Upper-limb motor recovery post-stroke is of special concern because the impact of UE impairments on disability and health is so marked [2, 3]. The persistence of UE impairments and the resultant inability to use the arm and hand prevents many individuals from returning to work, representing significant socioeconomic impact on the individual, the healthcare systems and society at large. While these problems are well recognized, little progress has been made in demonstrating substantive UE recovery in this population.

Repetitive transcranial magnetic stimulation (rTMS) is a form of noninvasive brain stimulation with the capacity to modulate cortical excitability. In health, transcallosal connections create an environment of mutual interhemispheric inhibition [4], critical to normal motor control, and especially relevant to performance of skilled movements. Following stroke, decreased corticospinal excitability in the lesioned hemisphere leads to an asymmetry in this mutual transcallosal inhibition [5, 6]. Decreased ipsilesional cortical excitability not only contributes directly to decreased drive to the corticospinal tract limiting activation of the contralateral musculature, but also produces an imbalance between the two hemispheres as inhibition to the contralesional hemisphere is compromised [7]. Following stroke, the contralesional M1 is argued to increase its inhibition acting on the ipsilesional M1, further reducing ipsilesional M1 excitability. During isolated voluntary movement of the paretic hand, the contralesional motor cortex imposes an abnormally high inhibitory drive onto the ipsilesional cortex [8] which may contribute to paretic hand impairment [9]. Downregulation of the contralesional hemisphere with a potential restoration of mutual interhemispheric inhibition may contribute to the restoration of paretic limb motor ability. Low-frequency (e.g., ≤1 Hz) rTMS inhibits regional brain activity and may increase contralateral cortical excitability via modulation of interhemispheric inhibition. In healthy controls, with the cerebral hemispheres functionally coupled and balanced, application of low-frequency rTMS to one hemisphere results in a decrease in the interhemispheric inhibition (IHI) from the stimulated to the unstimulated hemisphere [10–12].

Two recent meta-analyses concluded that rTMS produces positive effects on finger motor ability and hand function following stroke [13, 14]. Although these simple motor effects are certainly encouraging, real-world functional UE use requires engagement of the proximal shoulder and elbow to transport the distal effector as it engages with the environment. The purpose of this study was to evaluate, in the presence of chronic stroke, the effects of low-frequency rTMS to the contralesional hemisphere as an adjuvant to functional task practice, on UE functional ability. In light of the importance of functional, real world UE use we chose the Wolf Motor Function Test (WMFT) as our primary outcome as it provides an assessment of arm ability in its totality.

## 2. Material and Methods

### 2.1. Design

This double-blind randomized sham-controlled trial examined the immediate and retention effects of rTMS as an adjuvant to functional task practice of UE motor ability.

### 2.2. Subjects

Participants were identified through a University of Florida Institutional Review Board (UF IRB-01) approved registry at the Malcom Randall VA Brain Rehabilitation and Research Center (BRRC). Individuals who met the following inclusion criteria were invited to participate: (1) stroke onset > 6 months prior, (2) passive range of motion of paretic UE within functional limits, (3) Upper Extremity Fugl-Meyer Motor (UEFM-M) [15] assessment shoulder/elbow subcomponent score between 15–25 and (4) 18–90 years of age. Potential participants were excluded if they met any of the following criteria: (1) history of epilepsy, brain tumor, learning disorder, intellectual or developmental disabilities, drug or alcohol abuse, dementia, major head trauma, or major psychiatric illness, (2) history or radiographic evidence of arterio-venous malformation, intracortical hemorrhage, subarachnoid hemorrhage, or bilateral cerebrovascular disease, (3) history of implanted pacemaker or medication pump, metal plate in skull, or metal objects in the eye or skull, (4) use of medications known to lower seizure threshold, (5) pregnancy, (6) pain in paretic UE that would interfere with movement, (7) inability to follow 3-step instructions, (8) orthopedic condition in back or upper extremity, (9) impaired corrected vision that would alter kinematics of reaching, or (10) previous exposure to rTMS. Individuals who met these criteria provided written, signed Informed Consent approved by the UF IRB-01 prior to enrollment. Participants were randomly assigned to either the experimental (EXP) group to receive real-rTMS or the control (CON) group to receive sham-rTMS. Randomization was performed using a random number generator constrained to produce an equal number of participants in each group.

### 2.3. Intervention

Each (*n* = 16; 4 times per week for 4 weeks) intervention session consisted of low-frequency rTMS (real or sham) to the contralesional hemisphere followed by 1-hour of functional task practice activities, directed by a physical or occupational therapist, aimed at improving motor ability of the paretic arm and hand.

#### 2.3.1. rTMS Intervention

rTMS was delivered using a Magstim Rapid^2^ Stimulator (Magstim Company, Whitland, UK) with an air-cooled figure-8 coil (70 mm in diameter per loop) over the contralesional M1, focused over the optimal spot for stimulating the extensor carpi radialis (ECR) muscle. The ECR is important in attaining hand position for reach to grasp, and pointing to and touching objects in the environment, all of which are foundational movements for a functional upper extremity. First, the “hot spot,” the optimal scalp position for consistently eliciting the largest motor evoked potential (MEP) from the contralesional M1 representation corresponding to ECR, was determined. Next, resting motor threshold (rMT) was determined by decreasing the stimulus intensity over the hot spot. rMT was defined as the lowest stimulator output that elicited MEPs with a peak-to-peak amplitude of 50 *μ*V in 6 of 10 trials [16]. The center of the figure-8 coil was positioned tangentially over the “hot spot,” and oriented perpendicular to the central sulcus for optimal stimulation of the underlying tissue. A total of 1200 pulses were delivered as a single 1 Hz train, at 100% of rMT, established at each session. These stimulation parameters have been used in previous studies [17–19] and fall within the current recommended safety guidelines for rTMS [20]. Sham-rTMS was delivered using a placebo coil that looks like and imitates the sound of a real coil.

#### 2.3.2. Functional Task Practice

Intervention was delivered by a trained occupational or physical therapist, blinded to participants' group assignments. Session content was individualized to participants' interests, life roles, and level of motor function. All tasks were functional in nature and included reaching, grasping and/or manipulation of objects performed with the paretic UE only. Specific tasks were then chosen/designed by the interventionist for participants' to work at their challenge point. Examples of activities include manipulation of coins, keyboarding, tossing/catching, transporting items to/from various shelf heights, and opening closing drawers and doors. A detailed log of activities performed (e.g., time on task, total number of repetitions, repetitions/time) was recorded for each session to ensure that participant was being progressed at each session. At the subsequent session the interventionist would review the previous session's log to ensure progression of training parameters.

### 2.4. Outcome Measures

All outcome measures were assessed four times: pre-intervention baseline assessments were repeated 1-week apart, after intervention and at 30-day retention. Standardized assessors, who were physical therapists blinded to group assignment, conducted behavioral evaluations. Neurophysiologic and kinesiologic measurements were conducted by study investigators.

#### 2.4.1. Clinical Measures

A clinical assessment battery evaluated participants across the domains of the World Health Organization's International Classification of Function, Disability, and Health [21]. The Wolf Motor Function Test (WMFT) [22] was the primary behavioral outcome measure of arm function. Other measures included Grip, Lateral Pinch (LP), Palmar Pinch (PP), and 3-Jaw Chuck (3JC) force [23], UEFM-M [15] and the Action Research Arm Test (ARAT) [24]. Light Touch (LT) sensation of the volar surface of upper arm, palm, first digit and fifth digit was measured with Semmes-Weinstein Monofilaments (North Coast Medical, Morgan Hill, CA) [25]. The smallest monofilament sensed at each location was recorded and given an ordinal score, using a previously described scale [26]. The Modified Ashworth Scale (MAS) [27] was used to assess muscle tone (composite score of shoulder adductors and internal rotators, elbow flexors, forearm pronators, wrist flexors, and finger flexors). The Motor Activity Log (MAL) [28], a subjective assessment of the amount of use (MAL-AOU) and how well the arm and hand move (MAL-HW) in attempting real life tasks outside of the laboratory environment and the Late-Life Functioning Disability Index (LLFDI) [29], a comprehensive assessment of functional limitations (altered ability to perform specific actions encountered in daily routines) and disability (altered performance of major life tasks and social roles) were administered.

#### 2.4.2. Neurophysiologic Measures

Corticomotor excitability of the contralesional hemisphere was assessed by determining: (1) rMT of the unaffected ECR, (2) *V*_50_, the stimulus intensity at which MEP amplitude is 50% of the *MEP*⁡_MAX_, and (3) short intracortical inhibition (SICI). Surface electromyographic signals were acquired using bipolar Ag-AgCL electrodes applied in a belly-tendon montage over the ipsilesional ECR muscle. Signals were filtered between 1 and 1000 Hz, amplified, and digitized at a sampling rate of 2000 Hz. Data were acquired using a Powerlab16SP (AD Instruments, Grand Junction, CO), and stored for later offline analysis using LabChart Pro Software (Version 7.3.7). Single TMS pulses were delivered using a Magstim 200^2^ stimulator with figure-8 coil. The coil was oriented perpendicular to the central sulcus for optimal stimulation of the underlying motor cortex [30]. The “hotspot” corresponded to the stimulation site for which the largest MEPs were obtained in the contralateral ECR. 

*rMT.* Resting motor threshold was determined as the minimum stimulator intensity, expressed as a percentage of maximum stimulator output (% MSO), capable of eliciting a motor evoked response ≥50 *μ*V in at least 5 of 10 consecutive trials [16]. 

*Recruitment Curves. *Starting from rMT, stimulation intensity (SI) was increased in increments of 5% (MSO) with five stimuli applied at each SI until no further increase in MEP amplitude was observed. Individual stimuli were delivered in random intervals every 7–10 seconds to avoid stimulus anticipation.

*Short Intracortical Inhibition (SICI). *SICI was measured by paired-pulse (conditioned-test) stimulation with an inter-stimulus interval of 3 ms. SICI, a measure of inhibitory intracortical activity, reflects GABA_A_-ergic activity in cortical circuits [31, 32]. The test stimulus was delivered at the SI that produced a resting MEP of ~1 mV peak to peak. The conditioning stimulus was delivered at 70% of the test stimulus. Single and paired stimulations (*n* = 5 each) were delivered in random order. Responses within each condition (i.e., paired, single) were averaged. SICI was expressed as the ratio of conditioned MEP amplitude/test MEP amplitude where values <1 reflect inhibition and >1 reflect facilitation.

#### 2.4.3. Kinematics of Reaching

Participants were seated on a backless bench with feet flat on the floor and hips, knees, and ankles aligned at approximately 90 degrees of flexion. The start position of the upper extremity was defined as hands, palms side down, on the ipsilateral thigh with the shoulder at approximately zero degrees of flexion. The target was a 40 mm sphere positioned 90% of arm's length (measured from acromion process to tip of index finger), aligned with the reaching arm's shoulder joint height and lateral location. Participants were instructed to touch the target with their index finger of the affected UE as fast as possible, in response to a verbal “Go” signal. Participants practiced 1 to 2 trials prior to recording to familiarize themselves with the task and the instructions followed by 3 recorded trials.

Movements of the UE and trunk were captured by tracking the three-dimensional position of 14 mm reflective markers using an eight-camera (T40; 4 megapixel resolution) motion analysis system (Vicon Nexus 1.8.5 software, Oxford Metrics Inc., Oxford, UK) at a sampling frequency of 200 Hz. Marker placement is based on a modified version of the model described by Rab et al. and joint coordinate system defined by Wu et al [33, 34]. The markers were affixed to the following anatomical landmarks using double-sided tape: seventh cervical and tenth thoracic vertebrae, suprasternal notch, sternum body, sacrum, posterior superior iliac spine, anterior superior iliac spine, acromion process, medial and lateral epicondyles, styloid process of the radius and ulna, lateral end of the second and fifth metacarpal bones and distal phalange of the second digit. Stationary triads of noncollinear 9 mm reflective markers were affixed mid shaft to the upper and lower arms using Nylatex wraps and secured with Mueller foam pre-wrap.

*Data Reduction and Analysis.* Marker data were labeled, filtered (Butterworth, low-pass, 1st order, 10 Hz, bidirectional) and used to model (C-Motion, Visual3D software, Germantown, MD) three-dimensional shoulder, elbow and wrist joint angles. Additional analyses were performed using custom MATLAB code (R2011b, MathWorks, Natick, MA). Two events were manually identified using the Vicon Nexus software: start and touch. Start was the time of movement initiation and touch was the time when the index finger reached the target. The metrics of interest for this study were (1) movement time (MT), (2) maximum resultant velocity (MRV), (3) trunk displacement (TD), (4) shoulder range of motion (SROM), (5) elbow range of motion (EROM), and (6) wrist range of motion (WROM). Movement time was measured between the point where velocity surpassed 5% of peak velocity and the point at which it fell below 5% of peak velocity. Maximum resultant velocity was the point at which velocity was the greatest. Trunk displacement in the anterior-posterior direction during the reach task was measured with the marker placed at T10. Shoulder, elbow, and wrist range of motion are defined as the different the maximum and minimum values each joint achieved from start to touch in the flexion/extension direction.

Neurophysiologic data were analyzed using custom MATLAB code (R2011b, Mathworks, Natik, MA, USA). EMG data were filtered, demeaned, and rectified. MEPs at each SI were averaged and the mean MEP area was calculated from the averaged signal. MEP area at each SI was normalized to MEP area at motor threshold. Recruitment curves were constructed by plotting SI against MEP area followed by fitting with a nonlinear equation (Boltzmann sigmoid) to obtain the following parameters:

(1) $$\begin{array}{c} \mathrm{MEP}\left(s\right)=\text{Bottom}+\frac{\left(\text{Top}-\text{Bottom}\right)}{1+\exp\left(\left(V_{50}-x\right)/\text{Slope}\right)}, \end{array}$$

where *s* represents SI, Top represents the peak height of the recruitment curve, and *V*_50_ represents the SI at which the MEP amplitude is 50% of the *MEP*⁡_MAX_ [35, 36]. *V*_50_ was used as the dependent variable to represent cortical excitability.

### 2.5. Statistical Analyses

Study sample size was determined from previously published data on our primary outcome measure, the Wolf Motor Function Test [37]. In that study of 224 poststroke individuals the average change on the WMFT was 9.16 seconds. Anticipating a 50% greater improvement in the contralesional rTMS + behavioral training than the sham rTMS + behavioral training, a sample size of 10 in each group was determined to provide 80% power with a *P* > 0.05. Both time to complete and movement quality of each WMFT task were assessed. The average time was computed for all 15 tasks. Due to its skewed distribution (i.e., maximal time = 120 seconds) WMFT-T scores were treated using the natural logarithmic transformation to approximate a normal distribution [37, 38]. Tabled logs were converted back to seconds to aid interpretation. The Functional Assessment Scale (WMFT-FAS), a 0–5 rating of movement quality (0 = movement not attempted; 5 = movement appears normal) was summed across the 15 items. Baseline health and demographic characteristics were summarized (by mean ± SE or proportion) and compared across treatment groups using the Kruskal-Wallis test (for continuous variables) or an exact chi-square test (for categorical variables). The primary outcomes were tested using the change scores from preintervention to postintervention and from postintervention to the 1-month retention assessment. Each outcome was summarized (by mean ± SE) and compared between treatment groups (real-rTMS versus sham-rTMS) using the Wilcoxon rank sum test or two-sample *t*-test depending on the corresponding data distribution. The signed rank test or paired *t*-test was used for assessing the within-treatment change in each variable and the overall change based on the pooled data from both treatment groups. In addition, analysis of covariance (ANCOVA) was used to assess adjusted treatment effects for relevant variables controlling for baseline differences between groups. All analyses were performed using SAS 9.3 (SAS Institute Inc., Cary, NC).

## 3. Results

### 3.1. Recruitment

**Figure 1:**
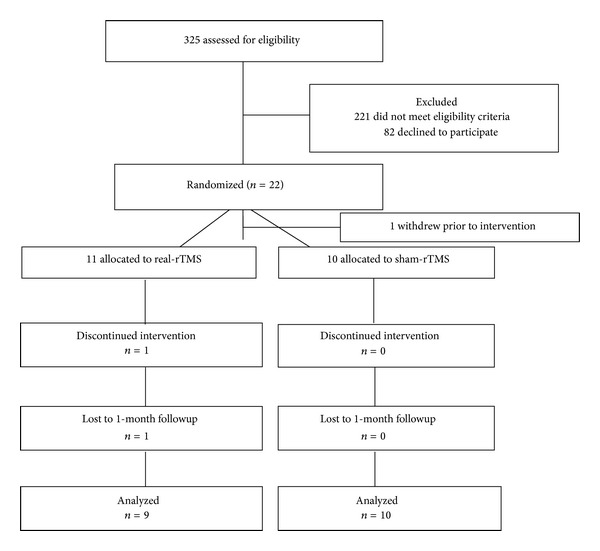
Flow of participants through the trial according to the CONSORT statement (rTMS, repetitive transcranial magnetic stimulation).

From three hundred twenty-five individuals screened 104 met eligibility criteria. The most common reasons for exclusion were (1) comorbid medical conditions (*n* = 98), (2) no overt motor deficit of the contralesional UE (*n* = 56), and (3) excessive motor deficit of the contralesional UE (*n* = 44). Eighty-two individuals were eligible but declined to participate secondary to lack of transportation, distance to study site, or time commitment required. Twenty-two individuals were randomized (Figure 1).

### 3.2. Participant Flow and Characteristics of Participants

Of the 22 subjects randomized, 1 withdrew prior to the start of intervention, 1 withdrew during the intervention, and 1 withdrew following completion of the intervention but prior to the 1-month retention assessment. No differences were revealed in participants' age, time post-stroke, stroke location or race between the two groups (Table 1). An equal number of males and females were represented in the sham-rTMS group whereas the real-rTMS group had just one female participant (see Table 1). No baseline differences were revealed between groups in any of the clinical or kinesiologic outcome variables. Baseline differences in SICI and *V*_50_ were detected. SICI was higher (49.6 versus 27.2; *P* = 0.02) and *V*_50_ was lower (43.4 versus 60.9; *P* = 0.03) at baseline for the real-rTMS compared to the sham-rTMS group.

### 3.3. Intervention and Subject Compliance

Ten of 11 participants randomized to the real-rTMS group completed the intervention. One participant in this cohort was unable to return for the 1-month retention assessment. All 10 participants randomized to the sham-rTMS group completed all aspects of the study. There were no adverse events related to the rTMS or the behavioral intervention.

### 3.4. Outcome Measures: Motor Impairment

No significant differences were detected in either preintervention to postintervention or postintervention to retention change scores for any of the clinical measures across the ICF model between the real-rTMS and sham-rTMS cohorts (Table 2). Change scores were then pooled across both groups for each of the clinical measures to determine whether significant intervention-related changes could be detected with a larger sample. For the entire cohort of participants, significant changes from pre- to postintervention were detected in the following variables: WMFT-T (−0.18 ± 0.09; *P* = 0.05), WMFT-FAS (2.3 ± 0.84; *P* = 0.01), UEFM-M (4.2 ± 1.0; *P* = 0.001), Grip force (1.4 ± 0.64; *P* = 0.04), ARAT (1.7 ± 0.73; *P* = 0.03), MAS (−1.8 ± 0.49; *P* = 0.00), MAL-AOU (0.58 ± 0.18; *P* = 0.00), and MAL-HW (0.62 ± 0.16; *P* = 0.00) scales. No significant changes were detected between postintervention and retention for all outcome measures except for Grip force (−1.26 ± 0.51; *P* = 0.024) (Table 3).

### 3.5. Outcome Measures: Kinematic

No significant differences in preintervention to postintervention or postintervention to retention change scores were detected for any of the kinematic variables measured during the reach to point task between the real-rTMS and sham-rTMS cohorts (Table 2). There were no differences in these measures when the data were pooled across groups.

### 3.6. Outcome Measures: Neurophysiologic

**Figure 2:**
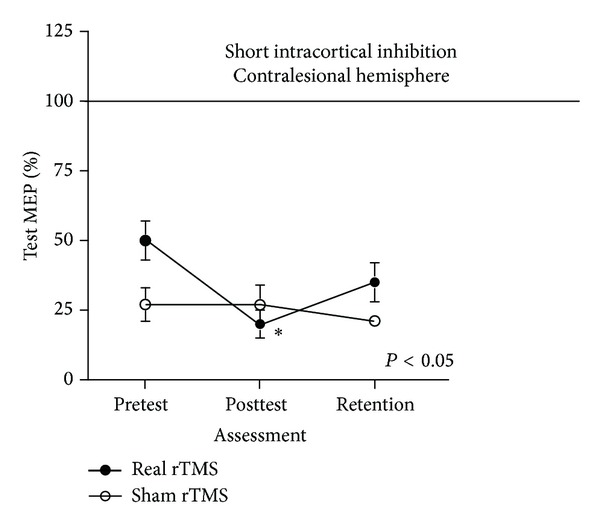
No change in contralesional hemisphere SICI following sham rTMS (open circle) in contrast to increased contralesional SICI (greater inhibition) for the real inhibitory rTMS group. (closed circle). Contralesional hemisphere SICI in the real rTMS group was significantly reduced, suggesting a downregulation of the preintervention hyperexcitable contralesional hemisphere. This downregulation observed in the real rTMS group at posttest was not retained at the 1-month retention assessment.

Significant between-group differences were detected in change scores for contralesional hemisphere SICI (−29.22 ± 7.56 versus −0.33 ± 4.18; *P* = 0.006) and *V*_50_ (3.75 ± 2.89 versus −6.85 ± 3.51; *P* = 0.034) from pre- to post-intervention (Figure 2). However, after adjusting baseline differences in these two variables between groups, no significant differences were detected for changes in either contralesional SICI (*P* = 0.066) or *V*_50_ (*P* = 0.344)_._

## 4. Discussion

This RCT evaluated the potential of low-frequency rTMS applied to the contralesional hemisphere as an adjuvant to functional task practice of the paretic UE to augment motor recovery in persons chronic poststroke. We designed the intervention dose (i.e., 1 hour session; 16 total sessions) to align with that observed in clinical practice. Contrary to our hypothesis we did not detect an augmentative effect of contralesional low-frequency rTMS in chronic UE hemiparesis with this design and these intervention parameters. In contrast 16 1-hour sessions of functional task practice produced small improvements in outcome measures at the impairment and functional limitation levels, regardless of whether it was preceded by sham or real 1 Hz rTMS applied over the hand area of contralesional M1. These results corroborate those of others who have reported no additional improvement in UE motor ability following stroke from the application of contralesional rTMS [39–41]. Our results point to a trend towards a decrease in the contralesional hemisphere hyperexcitability as measured by SICI; however, this change in cortical physiology did not correspond to functional restoration of paretic UE motor ability. Although contralesional hyperexcitability may contribute to UE motor impairment, the lesioned hemisphere remains the primary contributor to these deficits. Modulation of cortical excitability may not be sufficient to contribute to paretic UE motor ability in the presence of the primary lesion.

Administration of low-frequency rTMS for 20 minutes, 4 times/week followed by 1 hour of affected UE functional task practice was both feasible and well-tolerated by all participants with no adverse events from either aspect of the intervention. Previous studies of contralesional rTMS have examined effects following only a single bout of rTMS [19, 42–46] or repeated bouts but fewer than the number studied here [18, 39–41, 47]. The one study that administered a greater number of sessions [48] did so within the confines of an inpatient rehabilitation admission, easing participant burden.

Study participants were, on average, greater than 5 years from stroke onset. Although most investigations of rTMS enroll individuals greater than 6 months following stroke, participants in this study exceeded the chronicity of other stroke participant cohorts. It is possible that the interventions employed in this study (both neurophysiological and behavioral) were not able to induce change in individuals with the degree of chronicity represented in this study.

For a new modality to be adopted into clinical practice, a functional benefit must be observed. We purposely chose a multijoint functional measure, the WMFT, as our primary outcome measure to ascertain if functional changes in arm ability that could be adopted into everyday life would result from this combined intervention of low-frequency rTMS and functional task practice. In contrast to other studies that reported improvements in simple movements of the distal aspects of the effector, low-frequency rTMS combined with functional task practice did not improve functional, whole-arm movements more than control sham-rTMS.

*Effects on Contralesional M1 Cortical Excitability*. Following adjustments for baseline differences between the two groups there were no differences in the changes in contralesional M1 cortical excitability following the intervention. These results stand in contrast to previous studies that have reported differences in cortical excitability following application of contralesional low-frequency rTMS [44, 45]. However, a recently conducted meta-analysis [13] of 8 studies reported that changes of neurophysiologic measurements were not significant, although the trend of these changes was positive, as were ours. Our participants were more chronic poststroke than participants in studies that have reported modulation of cortical excitability following rTMS. The role of chronicity in cortical excitability responsiveness to this modality is not known and is an avenue of exploration that is needed. 

*Effects on UE Functional Ability*. Despite positive and expected changes in SICI (e.g., reduced disinhibition) following application of low-frequency rTMS, this postintervention change was not associated with greater improvement in paretic UE behavioral function for the experimental as compared to the control group. However, when data from both groups were pooled, small but statistically significant improvements were revealed in measures of arm impairment and function. Additionally these improvements were maintained for at least one month following the conclusion of the intervention, even in the presence of chronic stroke. We intentionally designed the time dedicated to the behavioral intervention to mirror which is realistically available in clinical practice. This limited amount of practice is probably insufficient to produce transformative changes in chronic UE functional limitations due to stroke.

## 5. Limitations

Analysis of baseline differences between groups revealed an unanticipated difference in contralesional motor cortex SICI between groups, precluding a straightforward between-group comparison. Secondly, we powered this study based on published data from the Extremity Constraint-Induced Therapy Evaluation (EXCITE) clinical trial [37]. The EXCITE data may not have been an appropriate reference as participants in the trial presented here were more chronic and received a smaller dose of behavioral intervention than EXCITE participants. Although there was a positive behavioral effect for the entire cohort, we may have been overoptimistic for this small, preliminary trial to detect group differences.

## 6. Conclusions

Although the contralesional hemisphere revealed somewhat greater intracortical inhibition following the intervention, changes in this variable alone were not sufficient to affect larger joint functional UE movements. Disinhibition of the contralesional cortex is one factor contributing to impaired UE motor ability following stroke. The primary stroke insult and the resultant decreased descending drive to the UE motor neuronal pools are not directly remediated by contralesional low-frequency rTMS and thus may be greater contributors to limb paresis. Although interhemispheric balance may be reestablished through downregulation of the hyperexcitable contralesional hemisphere by rTMS, further examination of its role in addressing poststroke UE impairment is needed.

## Figures and Tables

**Table 1:** Demographics of the study cohort.

Characteristic	rTMS group (*n* = 9)	Sham group (*n* = 10)
Mean age ± SD (y) (range)	64.7 ± 7.0 (55–74)	64.6 ± 9.0 (49–77)
Mean time from stroke onset ± SD (mo) (range)	60.4 ± 47.2 (9–146)	62.8 ± 51.7 (7–150)
Lesioned hemisphere	6 Left, 3 Right	4 Left, 6 Right
Sex	8 M, 1 F	5 M, 5 F
Race	9 W; 0 AA	9 W; 1 AA
UEFM-M score	37.5 ± 7.0	40.7 ± 11.6

AA: African American; M: male; F: female; SD: standard deviation; y: years; mo: months; UEFM-M: upper-extremity Fugl-Meyer motor.

**Table 2:** Changes in outcome measures from preintervention to postintervention and postintervention to retention by group: real-rTMS versus sham-rTMS.

Outcome measure	Postintervention-preintervention			Retention-postintervention		
	Within group change scores		Comparison between groups	Within group change scores		Comparison Between groups
	Real-rTMS mean (SE)	Sham-rTMS mean (SE)	Difference of change (95% CI)	Real-rTMS mean (SE)	Sham-rTMS mean (SE)	Difference of change (95% CI)
WMFT-*T* (log performance time)	−0.14 (0.14)	−0.21 (0.10)	0.07 (−0.30–0.44)	−0.008 (0.08)	0.13 (0.12)	−0.14 (−0.45–0.17)
WMFT-*T* (seconds)	0.87 (1.2)	0.81 (1.1)	0.06 (−0.30–0.44)	0.99 (1.1)	1.1 (1.1)	−0.11 (−0.45–0.17)
WMFT-FAS	1.5 (1.1)	3.1 (1.3)	−1.55 (−5.09–1.99)	−1.3 (1.3)	−1.4 (1.4)	0.15 (−3.96–4.26)
UEFM-M	4.6 (1.5)	3.9 (1.5)	0.65 (−3.85–5.15)	−1.6 (0.80)	0.6 (1.5)	−2.16 (−5.90–1.59)
Grip force (kg)	1.2 (1.1)	1.6 (0.67)	−0.38 (−3.18–2.42)	−1.6 (1.0)	−0.98 (0.33)	−0.60 (−3.0–1.9)
LP force (kg)	0.08 (0.28)	0.51 (0.33)	−0.43 (−1.4–0.49)	−0.08 (0.46)	−0.26 (0.20)	0.18 (−0.93–1.28)
PP force (kg)	0.11 (0.19)	0.42 (0.35)	−0.32 (−1.189–0.56)	−0.36 (0.2)	−0.02 (0.19)	−0.33 (−0.91–0.25)
3JC force (kg)	0.10 (0.56)	0.46 (0.44)	−0.36 (−1.85–1.14)	−0.44 (0.33)	−0.21 (90.33)	−0.23 (−1.23–0.77)
ARAT	0.89 (1.2)	2.5 (0.89)	−1.56 (−4.71–1.59)	0.38 (91.5)	−0.40 (0.82)	0.78 (−2.9–4.5)
Modified Ashworth Scale	−2.0 (0.83)	−1.6 (0.02)	−0.40 (−2.54–1.74)	0.22 (0.66)	−0.60 (0.65)	0.82 (−1.1–2.8)
Light Touch	−0.70 (0.51)	−0.25 (0.69)	−0.45 (−2.26–1.36)	−0.11 (0.72)	0.4 (0.4)	−0.51 (−2.29–1.27)
MAL-AOU	0.71 (0.31)	0.45 (0.18)	0.27 (−0.5–1.03)	−0.09 (0.21)	−0.20 (0.14)	0.11 (−0.44–0.66)
MAL-HW	0.76 (0.30)	0.48 (0.15)	0.28 (−0.44–0.99)	−0.21 (−0.25)	−0.27 (0.14)	0.06 (−0.57–0.68)
LLFDI—function total	2.4 (1.2)	0.34 (0.93)	2.0 (−1.18–5.2)	−2.39 (0.99)	−0.19 (0.79)	−2.20 (−4.92–0.52)
LLFDI—upper extremity	3.8 (3.2)	4.3 (2.2)	−0.51 (−8.7–7.7)	−4.22 (3.48)	−1.48 (2.08)	−2.75 (−11.61–6.12)
LLFDI—frequency dimension total	1.4 (1.2)	0.34 (0.94)	1.1 (−2.1–4.2)	0.34 (1.56)	0.48 (1.05)	−0.14 (−4.21–3.94)
LLFDI—social role	2.0 (1.3)	0.20 (1.3)	1.8 (−2.0–5.7)	−0.16 (2.77)	0.31 (1.77)	−0.47 (−7.62–6.68)
LLFDI—personal role	2.2 (2.8)	1.9 (2.1)	0.34 (−7.1–7.8)	2.22 (2.01)	1.19 (3.17)	1.02 (−6.98–9.02)
LLFDI—limitation dimension total	2.1 (1.1)	3.6 (1.9)	−1.5 (−6.3–3.4)	−0.59 (2.34)	−1.0 (2.64)	0.40 (−7.08–7.89)
LLFDI—instrumental role	4.3 (2.0)	3.8 (2.2)	0.56 (−5.7–6.8)	−0.98 (2.41)	−1.32 (2.92)	0.34 (−7.69–8.37)
LLFDI—management role	5.5 (3.0)	4.2 (1.7)	1.3 (−6.2–8.8)	−2.68 (5.53)	−2.22 (3.54)	−0.45 (−14.72–13.82)

Movement time (sec)	−0.14 (0.08)	−0.10 (0.11)	−0.042 (−0.33–0.24)	0.08 (0.06)	−0.003 (0.09)	0.09 (−0.15–0.32)
Trunk displacement (mm)	−3.9 (5.9)	−2.1 (4.7)	−1.8 (−17.63–13.98)	7.2 (7.0)	−5.1 (6.1)	12.37 (−7.24–32.0)
Maximum resultant velocity (m/s)	0.07 (0.04)	0.12 (0.03)	−0.05 (−0.16–0.06)	0.001 (0.05)	0.00 (0.03)	−0.003 (−0.133–0.127)
Shoulder ROM (degrees)	−2.55 (2.39)	−0.2 (1.84)	−1.11 (−6.70–4.48)	6.00 (3.11)	1.33 (2.40)	4.67 (−3.72–13.05)
Elbow ROM (degrees)	1.25 (1.99)	0.7 (1.75)	−3.19 (−8.24–1.85)	0.44 (2.10)	−4.89 (2.11)	5.33 (−0.97–11.64)
Wrist ROM (degrees)	−2.00 (1.60)	−0.2 (0.98)	−6.94 (−18.43–4.54)	1.00 (1.52)	−5.44 (5.26)	6.44 (−5.87–18.76)

SICI (% of test MEP)	−29.22 (7.6)	−0.33 (4.2)	−28.9∗ (−47.6–10.1)	13.29 (6.3)	−6.1 (6.8)	19.34 (−0.39–39.19)
*V*_50_ (% MSO)	3.8 (2.9)	−6.9 (3.5)	10.6∗ (0.89–20.3)	−1.7 (6.8)	3.8 (4.7)	−5.5 (−24.0–13.1)
rMT (% MSO)	3.6 (3.0)	−7.9 (5.3)	11.5 (−1.7–24.7)	−4.0 (4.3)	1.7 (7.3)	−5.7 (−24.1–12.7)

*P < 0.05.

SE: standard error; CI: confidence interval; rTMS: repetitive transcranial magnetic stimulation; WMFT: Wolf Motor Function Test; FAS: Functional Assessment Scale; *T*: Time; UEFM-M: upper extremity Fugl-Meyer motor; LP: Lateral Pinch; PP: Palmer Pinch; 3JC: Three Jaw Chuck; ARAT: Action Research Arm Test; MAL: Motor Activity Log; AOU: Amount of Use; HW: How Well; LLFDI: Late Life Function and Disability Index; ROM: Range of Motion; sec: seconds; mm: millimeters; m/s: meters per second; SICI: short intracortical inhibition; MEP: motor evoked potential; % MSO: percentage of maximum stimulator output; rMT: resting motor threshold.

**Table 3:** Outcome measures for entire cohort (*n* = 20) from preintervention to postintervention and postintervention to retention.

Outcome measure	Preintervention	Postintervention	Retention
	Mean (SE)	Mean (SE)	Mean (SE)
WMFT-*T* (log performance time)	2.5 (0.27)	2.3 (0.27)∗	2.3 (0.29)
WMFT-*T* (seconds)	12.2 (1.3)	10.0 (1.3)∗	10.0 (1.3)
WMFT-FAS	46.2 (2.6)	48.5 (2.7)∗	47.6 (2.7)
UEFM-M	39.1 (2.1)	43.3 (2.4)∗	43.3 (2.4)
Grip force (kg)	14.0 (1.7)	15.4 (1.6)∗	14.2 (1.7)∗
LP force (kg)	4.8 (0.43)	5.2 (0.44)	5.0 (0.48)
PP force (kg)	2.8 (0.37)	3.0 (0.41)	2.9 (0.40)
3JC force (kg)	3.0 (0.52)	3.4 (0.56)	3.4 (0.50)
ARAT	32.0 (2.9)	33.7 (2.7)∗	33.7 (2.9)
Modified Ashworth Scale	5.3 (0.62)	3.5 (0.52)∗	3.2 (0.55)
Light Touch	3.8 (0.63)	3.3 (0.67)	3.4 (0.51)
MAL-AOU	1.3 (0.21)	1.9 (0.23)∗	1.8 (0.28)
MAL-HW	1.4 (0.22)	2.0 (0.24)∗	1.8 (0.30)
LLFDI—function total	45.6 (1.5)	46.9 (1.5)	46.2 (1.9)
LLFDI—upper extremity	49.4 (2.0)	53.4 (2.1)	51.3 (2.6)
LLFDI—frequency dimension total	47.2 (1.7)	48.0 (1.9)	48.5 (2.4)
LLFDI—social role	44.4 (2.3)	45.5 (2.7)	46.2 (3.1)
LLFDI—personal role	48.2 (2.4)	50.2 (3.1)	50.9 (4.3)
LLFDI—limitation dimension total	57.9 (1.9)	60.8 (1.9)	60.6 (2.7)
LLFDI—instrumental role	54.8 (2.6)	58.9 (2.2)	58.3 (2.9)
LLFDI—management role	74.5 (2.6)	79.3 (3.2)	77.7 (3.7)

Movement time (sec)	1.2 (0.11)	1.1 (0.098)	1.1 (0.10)
Trunk displacement (mm)	49.3 (6.8)	46.3 (6.7)	47.1 (6.0)
Maximum resultant velocity (m/s)	0.99 (0.08)	1.1 (0.087)	1.1 (0.093)
Shoulder ROM (degrees)	41.2 (3.1)	39.3 (3.3)	41.5 (3.6)
Elbow ROM (degrees)	10.9 (1.5)	13.9 (2.1)	12.5 (2.1)
Wrist ROM (degrees)	9.3 (2.0)	11.3 (2.9)	8.1 (2.1)

SICI (% of test MEP)	38.4 (5.2)	23.6 (4.1)∗	26.9 (3.6)
*V*_50_ (% MSO)	52.6 (4.2)	50.8 (3.1)	52.8 (4.0)
rMT (% MSO)	42.6 (3.3)	40.5 (4.4)	39.5

*P < 0.05.

SE: standard error; WMFT: Wolf Motor Function Test; FAS: Functional Assessment Scale; *T*: time; UEFM-M: upper extremity Fugl-Meyer motor; LP: lateral pinch; PP: palmer pinch; 3JC: three jaw chuck; ARAT: Action Research Arm Test; MAL: Motor Activity Log; AOU: amount of use; HW: how well; LLFDI: Late Life Function and Disability Index; ROM: range of motion; sec: seconds; mm: millimeters; m/s: meters per second; SICI: Short intracortical inhibition; MEP: motor evoked potential; % MSO: percentage of maximum stimulator output; rMT: resting motor threshold.
